# The Effects of General Mental Health Symptomatology, COVID Anxiety, and Sociodemographic Factors on Pandemic-Induced Negative and Positive Trauma Effects: A Polish Survey from the Late Stages of the Pandemic

**DOI:** 10.3390/jcm14103343

**Published:** 2025-05-12

**Authors:** Jakub F. Juranek, Maja Wojtkiewicz, Judyta K. Juranek, Jarosław Szuszkiewicz, Marcin Jóźwik, Joanna Wojtkiewicz

**Affiliations:** 1 Institute of Psychology, Polish Academy of Sciences, Jaracza 1, 00-378 Warsaw, Poland; jakub.juranek@sd.psych.pan.pl; 2 Department of Geomatics and Cartography, Faculty of Earth Sciences and Land Management, Nicolaus Copernicus University, 87-100 Toruń, Poland; maja.wojtkiewicz@umk.pl; 3 Department of Human Physiology and Pathophysiology, School of Medicine, Collegium Medicum, University of Warmia and Mazury, 10-045 Olsztyn, Poland; judyta.juranek@uwm.edu.pl; 4 Department of Materials Technology and Machinery, Faculty of Technical Sciences, University of Warmia and Mazury, 10-900 Olsztyn, Poland; 5 Department of Gynecology and Obstetrics, School of Medicine, Collegium Medicum, University of Warmia and Mazury in Olsztyn, 10-045 Olsztyn, Poland; marcin.jozwik@uwm.edu.pl

**Keywords:** anxiety, COVID-19, demographic factors, mental health, trauma

## Abstract

**Background/Objectives:** COVID-19, caused by the SARS-CoV-2 virus, emerged in late 2019 and rapidly became a global pandemic, affecting over 200 countries. The pandemic has had profound impacts on global health, the economy, and mental health, leading to increased anxiety and more cases of posttraumatic stress disorder in the general population. This study aimed to evaluate the factors influencing the long-term psychological effects, both positive and negative, seen in the late stages of the COVID-19 pandemic in Poland (starting in the second half of 2021). It combined specific COVID-19 anxiety factors with a broader general mental health assessment to identify significant associations. **Methods:** Data were collected from 416 participants through paper-based and online questionnaires, with 235 valid responses gathered in total. This study utilized the Coronavirus Anxiety Scale; General Health Questionnaire (GHQ-28); and, to study Positive Trauma Effects (PTEs) and Negative Trauma Effects (NTEs), the Changes in Outlook Questionnaire. Data analysis was performed using the R language and a Generalized Additive Model analysis was also performed. **Results:** The study found generally low levels of COVID-19 anxiety and mental distress among participants. Significant predictors of NTEs included COVID-19 anxiety and general mental health status, which explained 47% of the variance. PTEs were significantly associated with gender, with women experiencing higher PTE levels relative to men. **Conclusions:** The findings indicate that combining specific ailment anxiety measurements with general mental health assessments enhances our ability to predict Negative Trauma Effects. Addressing mental health symptomatology and well-being during mass health crises is crucial to mitigate long-lasting psychological damage.

## 1. Introduction

COVID-19, caused by the SARS-CoV-2 virus, emerged in China in late 2019 and quickly became a global pandemic by 2020, affecting over 200 countries and territories. The pandemic has profoundly impacted global health, economies, education, and communication and has necessitated unprecedented public health measures [1]. COVID-19 primarily affects the respiratory system but can also damage other organs, leading to long-term health issues such as organ damage, inflammation, fatigue, and mental health disorders like depression, anxiety, and posttraumatic stress disorder [2,3,4,5]. The pandemic has also exacerbated pre-existing chronic conditions and disrupted essential health services, disproportionately affecting vulnerable populations [6]. The pandemic has introduced a novel form of anxiety specific to COVID-19, which has been linked to adverse outcomes in both children and adults and has a particularly large impact on young people [7,8,9,10]. All members of the public, including healthcare professionals, showed significant levels of stress and anxiety due to the fear of infection and the possibility of transmission through family members and friends [11]. High levels of COVID-19 anxiety have been associated with increased mental health problems [9,10,11,12,13]. While intense stress and trauma typically result in adverse psychological effects, research has also shown that such events can facilitate positive psychological changes, a concept known as posttraumatic growth (PTG) [14]. Examples of PTG changes in people include a deeper appreciation for life, improved relationships, finding new opportunities, increased personal strength, and spiritual transformation [15]. The COVID-19 pandemic, bringing an acute threat to physical health, increased stress levels, and heightened exposure to psychologically painful events (like the illness and deaths of relatives, friends, and acquaintances), can be considered a significant source of adversity with high traumatic potential [16,17], an assumption which is confirmed by studies reporting posttraumatic symptoms in both COVID-19 patients and the general population [18,19]. The aim of this study was to evaluate the factors influencing the long-term psychological effects—both positive and negative—of the COVID-19 experience. This study is grounded in the posttraumatic growth (PTG) framework introduced by Tedeschi and Calhoun [15]. The PTG framework provides a theoretical lens for understanding how the interplay between individual characteristics and stressors can shape both adverse and adaptive responses to adversities, aligning with this study’s focus on the negative and positive psychological outcomes of the pandemic. The potential associations between specific COVID-19 anxiety factors were combined with a broader, general mental health assessment. Additionally, data depicting features of the late stages of the pandemic in a Polish sample were collected and used to unravel any potential significant associations between factors, such as vaccination and demographic variables.

## 2. Materials and Methods

### 2.1. Study Design

This study employed a cross-sectional, non-probability convenience sampling strategy to examine the relationships between mental health, COVID-19 anxiety, and trauma. The non-probability approach was chosen due to logistical challenges experienced during the COVID-19 pandemic, including restrictions on mobility and face-to-face interactions, which limited the feasibility of probability sampling. Data were collected via paper-based and online surveys to maximize participation, while acknowledging that this method prioritizes accessibility over strict representativeness.

### 2.2. Participants

A total of 416 participants completed the survey, with 318 responses obtained from the paper-based survey and 98 from the online survey. The data were collected from June to October 2021. After excluding 181 responses due to missing data or errors, the final sample consisted of 235 participants either with or without a history of COVID-19 illness. The sample included 173 females (73.6%) and 62 males (26.4%), which is a significant gender imbalance. This imbalance likely reflects the demographics of the sampled locations and platforms. The University of Warmia and Mazury, and particularly its medical and health sciences programs, has a higher proportion of female students and staff, which may have contributed to the overrepresentation of females in the paper-based survey. Similarly, the online survey, distributed through social media, may have attracted more female participants, as studies suggest that women are more likely to engage in health-related online surveys [20,21]. Additionally, the University Hospital, where part of the paper-based survey was conducted, serves a patient population that may include more females due to increased healthcare-seeking behavior among women [22].

The sample was predominantly urban, with 50.6% of participants residing in cities with populations of between 50,000 and 500,000, 20.4% in smaller towns (with up to 50,000 inhabitants), 6.8% in cities with over 500,000 inhabitants, and only 22.1% in rural areas (Table 1). The participants’ ages ranged from 16 to 83 years (mean = 37.03, median = 34). Most participants were single (49.8%), lived in medium-sized (up to a population of 500,000) cities (50.6%), and had completed secondary education (51.1%). The study was approved by the University Institutional Ethics Committee (approval number 20/2021, date of approval: 21 June 2021), and all participants provided their informed consent.

### 2.3. Materials

The questionnaires and information gathered in this study comprised the following:

1. Demographic Information: Age, gender, marital status, place of residence, education level, questions about whether participants had children and grandchildren, and personal or familial experience with COVID-19.
2. Coronavirus Anxiety Scale (CAS): A self-reported measure assessing anxiety related to the coronavirus pandemic, which consisted of five items rated on a 5-point Likert scale [23,24].
3. General Health Questionnaire (GHQ-28): This tool assesses general mental health and somatic symptoms. It consists of 28 items divided into four subscales: somatic symptoms, anxiety and insomnia, social dysfunction, and severe depression [25].
4. Changes in Outlook Questionnaire (CiOQ): A measure of positive and negative existential changes following a traumatic event, with a focus on questions related to the COVID-19 pandemic [26,27].

### 2.4. Data Analysis

Data were analyzed using the R language (version 4.3.1) with RStudio software. The “mgcv” library was used for Generalized Additive Model (GAM) analysis.

#### Statistical Techniques

Numerical variables, such as age, Coronavirus Anxiety Scale (CAS) scores, General Health Questionnaire (GHQ-28) subscale and overall scores, and the Changes in Outlook Questionnaire (CiOQ)’s positive (PTE) and negative (NTE) trauma effect scores, were expressed as means and standard deviations (SDs) for descriptive statistics. For example, age was reported as a mean of 37.03 (SD = 15.6), the CAS score as a mean of 1.16 (SD = 2.57), PTE as a mean of 18.27 (SD = 5.27), and NTE as a mean of 11.61 (SD = 4.61). Additionally, the minimum, maximum, median, skewness, and kurtosis were calculated for these variables to provide a comprehensive summary of their distributions, as shown in the descriptive statistics output.

Categorical variables, including gender, marital status, place of residence, education level, and binary variables (e.g., has_children, has_grandchildren, others_w_covid, others_hospitalized, others_dead, self_covid, self_vaccinated), were expressed as absolute numbers (N) and percentages (%). For instance, gender was reported as 173 females (73.6%) and 62 males (26.4%) and marital status was split into 117 single (49.8%), 89 married (37.9%), 21 divorced (8.9%), and 8 widowed (3.4%), as detailed in Table 1.

Statistical analyses used included Spearman correlations to assess the relationships between variables, with correlation coefficients (ρ) and *p*-values reported. Assumptions of linear regression (linearity, independence, homoscedasticity, normality, outliers, and influence) were evaluated as follows: linearity was assessed using a Residuals vs. Fitted plot, which showed a random scatter of points around the horizontal line at zero; independence was evaluated through Variance Inflation Factor (VIF) values, calculated using the car package and the Durbin–Watson test for the autocorrelation of residuals; homoscedasticity was checked with a Scale–Location plot to verify the constant variance of residuals; normality was verified using a Normal Q-Q plot to assess residual distribution; and outliers and influence were examined with a Residuals vs. Leverage plot and influence measures (Cook’s distance, DFFITS, and DFBETAS). Due to the presence of non-linear relationships, as indicated by diagnostic plots and tests (e.g., the Breusch–Pagan test for heteroscedasticity), the Generalized Additive Model (GAM) was selected for further analysis. The flexibility of GAM in capturing non-linear relationships without assuming a specific parametric form made it the most suitable choice for this study. All statistical tests were conducted with a significance level of *p* < 0.05.

## 3. Results

### 3.1. Descriptive Statistics

The results revealed that the majority of participants (85.8%) reported knowing someone who had contracted COVID-19, and 37% knew someone who was hospitalized due to COVID-19. Nearly a quarter (23.8%) had a family member, friend, or acquaintance who died of COVID-19, and 37% had contracted the virus themselves. Most respondents (83.4%) were vaccinated against COVID-19. The demographic characteristics of the study group are depicted in Table 1. Some level of COVID-19 anxiety has been self-reported in our study by 31.91% of the respondents, and the mean CAS score was 1.16 (SD = 2.57), indicating generally low levels of anxiety. The CiOQ results showed a mean PTE score of 18.27 (SD = 5.27) and a mean NTE score of 11.61 (SD = 4.61), suggesting moderate levels of both Positive and Negative Trauma Effects. The GHQ-28 results indicated low levels of mental distress, with mean scores ranging from 1.12 to 3.03 across subscales.

### 3.2. Correlation Analysis

The Spearman correlations revealed significant relationships in terms of the effects of trauma: NTEs were positively correlated with CAS (ρ = 0.38, *p* < 0.0001) and negatively correlated with education (ρ = −0.15, *p* < 0.05), and PTEs were negatively correlated with the male gender (ρ = −0.21, *p* < 0.01). All correlations are depicted in Table 2, Table 3 and Table 4.

### 3.3. Generalized Additive Model Analysis

Due to the non-linear nature of the data, the Generalized Additive Model (GAM) was used to analyze the relationships between the factors and trauma effects. The first model, intended to predict PTEs, explained 10.5% of the deviance, with gender as the only significant predictor. Female participants had higher PTE scores than male participants. The NTE model explained 47% of the deviance, with both the CAS (F = 25.05, *p* < 0.001) and GHQ Overall Score (F = 11.71, *p* < 0.001) showing significant relationships. The relationship between the GHQ Overall Score and NTEs was non-linear.

## 4. Discussion

Although this study has been conducted using a convenience sample, the participants were highly exposed to and aware of the pandemic, as evidenced by their high vaccination rate (83.4%) and widespread familiarity with the COVID-19 disease (85.1% knew someone who had contracted the virus). The demographics suggest this was a diverse but not necessarily representative sample of the Polish population. Our convenience sampling method prioritized accessibility over strict representativeness, which is a common trade-off in studies conducted during crises like the COVID-19 pandemic. This study found generally low levels of anxiety and mental distress among participants in the late stages of the pandemic, which is in contrast to reports from earlier stages of the pandemic [28,29,30,31,32]. This observed change may be due to several factors, such as a certain degree of habituation to the pandemic, the wide use and availability of vaccines, and improved institutional responses. The trauma effects observed were moderate, with COVID-19 anxiety and general mental health status being significant predictors of Negative Trauma Effects. Combined, those factors’ explanatory value for NTEs (at 42%) was much higher than that of COVID-19 anxiety alone, as previously reported in the literature [24], although caution needs to be applied to this comparison, as different analytical approaches have been used in both studies.

Recent studies using the meta-analytical approach [33,34] and a meta-analysis of meta-analyses [35] have highlighted the crucial role anxiety, depression, and other negative emotional states played in people’s experience of the pandemic. The percentage of our respondents reporting any level of anxiety standing at 31.91% is very similar to the percentages of around 30% shown in meta-analytical studies regarding general and specific populations (students, pregnant women, healthcare workers).

This study also found a significant association between gender and Positive Trauma Effects, with females experiencing higher levels of posttraumatic growth than males. This aligns with findings from other studies [36,37,38,39] (although some studies do not report any gender differences in this matter [40]), which may be attributed to the increased openness of women to sharing their experiences when affected by traumatic events, which facilitates cognitive processes that promote healthy reassessments [41]. In the context of the specifics of the COVID-19 pandemic, especially its widely imposed restrictions and lockdowns, it is also that suggested increased PTG might have stemmed from women being more family-oriented and finding more positive meaning in home and family activities [37]. While gender was identified as a significant predictor of posttraumatic growth (PTG), other psychological or sociodemographic factors, such as resilience or coping strategies, were not included in our investigation. These factors could provide deeper insights into the mechanisms driving positive trauma outcomes, as they are known to mediate responses to adversity [15]. Although, in our study, the gender difference was shown in the increased PTG in women, it is important to highlight the disproportionate psychological impact of the pandemic in terms of gender and other group factors, including age. Pre-existing mental health inequalities are exacerbated by health crises such as global pandemics, during which, for example, women and young people are reported to be at higher risk of anxiety and depression [42,43].

Additionally, in terms of its new contributions, our study valuably replicated some earlier reported associations, bolstering the validity of these findings and reinforcing their reliability. In our study, significant differentiation in COVID-19 infections by gender was observed, with men being less frequently infected than women. This has been reported in prior research, indicating a higher susceptibility to the infection but lower severity of the disease in women [44]. Age was associated with increased COVID-19 anxiety, likely due to older adults’ higher vulnerability to severe disease outcomes [45,46]. Residence in large urban areas was associated with higher GHQ mental health symptomatology scores compared to smaller urban and rural areas, consistent with previous findings on the adverse impact of urbanization on mental health [47,48,49].

It is important to highlight that the data recorded in our study from the use of the General Health Questionnaire tool relate to general symptomatology in mental health functioning, while the causes of the self-reported symptoms and their severity may stem, in the case of the COVID-19 health crisis, from different groups of factors, some of a situational and external nature and some of organic provenience, induced by the COVID-19 pathogen itself. Direct viral effects and chronic inflammation have been shown to lead to persistent mental health impairment in general and in the pandemic [50,51]. The neurotropic properties of COVID-19 lead, in some cases, to neurological manifestations, including mood disturbances, cognitive impairments, and heightened anxiety. Chronic inflammation triggered by the virus has been linked to depressive symptoms and increased vulnerability to psychiatric disorders. These biological factors, combined with pandemic-related stressors—such as social isolation, economic instability, and grief—create a complex interplay that exacerbates mental health outcomes [52]. Understanding these mechanisms is essential for developing targeted interventions that address both the physiological and psychological aspects of post-COVID-19 mental health recovery. Future research should explore integrated treatment approaches that consider neuroinflammation, trauma-informed care, and resilience-building strategies to mitigate long-term effects.

The long-term psychological effects of the COVID-19 pandemic extend beyond measurable anxiety and distress and encompass the consequences of social and school deprivation. Prolonged lockdowns, restricted social interactions, and disruptions to education likely fostered new behavioral habits, such as increased reliance on digital communication or altered social engagement patterns, which may continue to influence current functioning. For instance, social isolation during the pandemic has been linked to increased loneliness and reduced social skills, particularly among younger populations, with potential lasting impacts on mental well-being [53]. School closures disrupted academic progress and peer interactions, contributing to challenges in emotional regulation and social reintegration post-pandemic [54]. These effects may not manifest as elevated anxiety in the late stages of the pandemic, as observed in our study, but could subtly shape daily functioning and resilience.

Coping strategies likely played a critical role in fostering resilience and positive psychological outcomes, such as posttraumatic growth (PTG), during the COVID-19 pandemic. Within the posttraumatic growth framework [15], adaptive coping mechanisms, including seeking social support, engaging in meaning-making, and adopting problem-focused coping, may have facilitated PTG, particularly among the females who reported higher PTG in our study. For instance, social support from family or community networks has been shown to buffer stress and promote positive reinterpretations of traumatic experiences, contributing to enhanced resilience [55]. Similarly, adaptive behaviors, such as maintaining routines or finding purpose through creative or altruistic activities (e.g., volunteering, online community engagement), may have supported psychological growth despite pandemic-related adversities [56]. Our study did not directly measure coping strategies; the observed association between gender and PTG suggests that women may have utilized such strategies more effectively, potentially due to their greater emotional expressiveness or social connectedness. Future research should explore specific coping mechanisms to identify key resilience factors that mitigate the impact of catastrophic events and promote positive trauma outcomes.

Regarding vaccination, familiarity with someone hospitalized due to COVID-19 exhibited the strongest positive relation with being vaccinated, followed by knowing someone who died or fell ill with COVID-19. This is consistent with similar studies showing that personal exposure to severe COVID-19 outcomes increases the likelihood of vaccination [57,58]. Marital status (being married) was also found to be positively associated with vaccination, supporting most findings from other studies [59,60,61], although in this regard some contrasting relationships, possibly due to cultural and regional factors, exist [62]. Vaccination acceptance varies significantly between rural and urban populations due to differences in healthcare access, education levels, and exposure to public health campaigns. Studies have shown that urban residents generally exhibit higher vaccine uptake compared to rural populations. This disparity is often attributed to greater access to healthcare facilities, higher levels of health literacy, and increased exposure to pro-vaccine messaging in urban areas. Conversely, rural communities tend to experience lower vaccination rates, influenced by factors such as limited healthcare infrastructure, lower trust in medical authorities, and the prevalence of misinformation [63,64]. Our study did not investigate this difference due to the high percentage of the urban respondents.

Unlike previous research, we found no significant correlation between education level or gender and vaccination [65,66,67].

Future research should expand upon the findings of this study by investigating additional psychological and sociodemographic factors that contribute to posttraumatic growth and mental health outcomes in the aftermath of the pandemic. Resilience, coping strategies, and social support may be important factors shaping recovery after a pandemic. Addressing the issue of the mental health inequalities that manifested themselves during the pandemic requires an intersectional approach that considers economic and healthcare access. Longitudinal studies tracking the long-term psychological trajectories of the factors included in this study, enriched with additional variables, will be crucial in developing evidence-based interventions to mitigate lasting effects and improve public health responses to similar crises.

## 5. Limitations

This study has limitations that should be considered when interpreting its results. First, the sampling utilized may limit the representativeness of the investigated group with regard to the population. The sample was drawn from specific locations (e.g., the University of Warmia and Mazury campus and University Hospital) and online platforms, which may not fully reflect the broader Polish population. Notably, the sample exhibited a significant gender imbalance, with 73.6% of participants being female compared to 26.4% being male. This overrepresentation of females may have influenced the findings, particularly those regarding posttraumatic growth, as our results indicated that females experienced higher levels of PTG compared to males, although gender has been accounted for in the GAM as a predictor and our result is consistent with prior research [68]. Additionally, the reliance on self-reported measures carries the risk of response bias, as participants might under- or overestimate their anxiety and trauma effects based on personal perceptions, social desirability, or recall inaccuracies, potentially affecting the accuracy of the reported outcomes. Finally, our ability to generalize these findings to rural populations, who may have experienced unique stressors and coping mechanisms during the COVID-19 pandemic, may be limited. However, given this study’s focus on psychological patterns rather than geographic differences, this limitation does not undermine the validity of the core analyses.

## 6. Conclusions

This study explored the value of short, survey-based general mental health assessments combined with dedicated COVID-19 anxiety measurements for probing the association between Negative and Positive Trauma Effects and aspects of the pandemic. The findings indicate that combining measurements of specific ailment anxieties with general mental status assessments greatly increases our ability to predict Negative Trauma Effects, as mental health symptom status and COVID-19-related anxiety together explain almost half of the variance in NTEs. This indicates that decreased mental health and elevated anxiety are crucial factors contributing to NTE development. The same measurements, however, do not hold any predictive value for Positive Trauma Effects, for which the only significant associated variable detected was gender. The results of this study show that in addition to addressing the cause of the threat in a mass health crisis (i.e., the virus in the COVID-19 pandemic), additional attention directed toward mental health symptomatology and well-being in such events is crucial, especially attention that encompasses the levels below the thresholds signifying a pathology, in order to mitigate the negative effects of trauma and long-lasting damages to psychological health.

## Figures and Tables

**Table 1:** Characteristics of the study group (N = 235).

	M	SD
Age	37.03	15.6
	N	%
Sex		
Women	173	73.6
Men	62	26.4
Education		
Primary	4	1.7
Secondary	120	51.1
Tertiary	111	47.2
Residency		
Rural	52	22.1
City with up to 50,000 inhabitants	48	20.4
City with 50,000 to 500,000 inhabitants	119	50.6
City with over 500,000 inhabitants	16	6.8
Marital Status		
Single	117	49.8
Married	89	37.9
Divorced	21	8.9
Widow(er)	8	3.4
Children		
Yes	118	49.8
No	117	50.2
Grandchildren		
Yes	30	12.7
No	205	87.2
Knowing Someone with COVID-19		
Yes	200	85.1
No	35	14.9
Knowing Someone Hospitalized due to COVID-19		
Yes	87	37
No	148	63
Knowing Someone Deceased due to COVID-19		
Yes	56	23.8
No	179	76.2
Self-reported COVID-19 Infection		
Yes	87	37
No	148	63
Self-reported Vaccination Status		
Yes	196	83.4
No	39	16.6

**Table 2:** Correlations between trauma effects, demographic factors, COVID-19 experiences, and vaccination (N = 235).

	Positive Trauma Effect (PTE)	Negative Trauma Effect (NTE)
Sex	−0.21 **	−0.11
Age	0.13	−0.02
Coronavirus Anxiety Scale (CAS)	−0.03	0.38 ****
Marital Status	0.04	−0.01
Place of Residence	−0.08	0.09
Education Level	0.03	−0.15 *
Has Children	0.11	0
Has Grandchildren	0.06	−0.01
Knows Others with COVID-19	0.06	−0.11
Knows Others Hospitalized Due to COVID-19	0.09	−0.02
Knows Others Dead Due to COVID-19	0.12	−0.04
Self-Experienced COVID-19 Illness	0.07	−0.01
Vaccinated Against COVID-19	0.11	0.12

Sex (0—female; 1—male); age; CAS: Coronavirus Anxiety Scale; MS: marital status (0—single, divorced, or widowed; 1—married); PR: place of residence (0—village or city with up to 100,000 inhabitants; 1—city with over 100,000 inhabitants); EDU: education (0—below higher; 1—higher); CH: children (0—no children; 1—has children); GR: grandchildren (0—no grandchildren; 1—grandchildren); OC: others with COVID-19 (0—not knowing others that had COVID-19; 1—knowing others that had COVID-19); OH: others hospitalized due to COVID-19 (0—not knowing others hospitalized due to COVID-19; 1—knowing others hospitalized due to COVID-19); OD: others dead due to COVID-19 (0—not knowing others dead due to COVID-19; 1—knowing others dead due to COVID-19); SE: self-experienced COVID-19 illness (0—no; 1—yes); VAC: vaccination (0—no; 1—yes); PTE: Positive Trauma Effect; NTE: Negative Trauma Effect. **Note**: correlation coefficient = Spearman’s ρ. Significance levels: * *p* < 0.05, ** *p* < 0.01, **** *p* < 0.0001.

**Table 3:** Correlations between demographic factors, COVID-19 experiences, and vaccination (N = 235).

	Sex	Age	CAS	MS	PR	EDU	CH	GR	OC	OH	OD	SE
Age	0.07											
Coronavirus Anxiety Scale	−0.06	0.18 **										
Marital Status	0.07	0.50 ****	0.01									
Place of Residence	−0.05	0.11	0.17 **	−0.09								
Education	0.01	0.44 ****	0.03	0.30 ****	0.25 ***							
Children	0.06	0.77 ****	0.11	0.67 ****	−0.05	0.36 ****						
Grandchildren	0.00	0.55 ****	0.13 *	0.17 **	0.15 *	0.12	0.36 ****					
Others with Covid	−0.02	−0.12	−0.05	0.01	0.03	0.11	−0.06	−0.09				
Others hospitalized	−0.1	0.02	0.06	0.06	0.09	0.09	0.04	−0.06	0.32 ****			
Others dead	−0.06	0.14 *	0.06	0.1	0.00	0.13 *	0.16 *	0.00	0.23 ***	0.61 ****		
Self-experienced COVID-19	−0.16 *	−0.07	−0.02	0.00	−0.07	−0.05	−0.08	−0.06	0.27 ****	0.22 ***	0.13 *	
Vaccination	−0.04	0.05	0.05	0.16 *	−0.01	0.03	0.06	0.00	0.17 *	0.22 ***	0.20 **	0.01

Sex (0—female; 1—male); CAS: Coronavirus Anxiety Scale; MS: marital status (0—single, divorced, or widowed; 1—married); PR: place of residence (0—village or city with up to 100,000 inhabitants; 1—city with over 100,000 inhabitants); EDU: education (0—below higher; 1—higher); CH: children (0—no children; 1—has children); GR: grandchildren (0—no grandchildren; 1—grandchildren); OC: others with COVID-19 (0—not knowing others that had COVID-19; 1—knowing others that had COVID-19); OH: others hospitalized due to COVID-19 (0—not knowing others hospitalized due to COVID-19; 1—knowing others hospitalized due to COVID-19); OD: others dead due to COVID-19 (0—not knowing others dead due to COVID-19; 1—knowing others dead due to COVID-19); SE: self-experienced COVID-19 illness (0—no; 1—yes); VAC: vaccination (0—no; 1—yes). Note: correlation coefficient = Spearman’s ρ. Significance levels: * *p* < 0.05, ** *p* < 0.01, *** *p* < 0.001, **** *p* < 0.0001.

**Table 4:** Correlations between General Health Questionnaire (GHQ-28) scores, demographic factors, COVID-19 experiences, and vaccination (N = 235).

	GHQ−A	GHQ−B	GHQ−C	GHQ−D	GHQ Overall
Sex	−0.07	−0.09	0.04	−0.05	−0.04
Age	0.06	−0.03	0.17 **	−0.08	0.04
COVID−19 Anxiety Scale (CAS)	0.26 ****	0.39 ****	0.37 ****	0.30 ****	0.36 ****
Marital Status	−0.09	−0.08	0.04	−0.05	−0.09
Place of Residence	0.22 ***	0.16 *	0.22 ***	0.13 *	0.23 ***
Education Level	0.09	0.01	0.1	0.02	0.04
Has Children	−0.06	−0.11	0.06	−0.1	−0.07
Has Grandchildren	0.08	0.00	0.15 *	0.02	0.09
Knows Others with COVID−19	0.13 *	0.12	0.00	0.10	0.10
Knows Others Hospitalized Due to COVID−19	0.08	0.08	−0.01	0.05	0.06
Knows Others Dead Due to COVID−19	0.03	0.00	−0.04	−0.01	−0.01
Self−Experienced COVID−19 Illness	0.09	0.04	−0.07	0.05	0.05
Vaccinated Against COVID−19	0.05	0.04	−0.02	−0.07	0.04

Sex (0 = female, 1 = male); age; CAS: Coronavirus Anxiety Scale; MS: marital status (0—single, divorced, or widowed; 1—married); PR: place of residence (0—village or city with up to 100,000 inhabitants; 1—city with over 100,000 inhabitants); EDU: education (0—below higher; 1—higher); CH: children (0—no children; 1—has children); GR: grandchildren (0—no grandchildren; 1—grandchildren); OC: others with COVID-19 (0—not knowing others that had COVID-19; 1—knowing others that had COVID-19); OH: others hospitalized due to COVID-19 (0—not knowing others hospitalized due to COVID-19; 1—knowing others hospitalized due to COVID-19); OD: others dead due to COVID-19 (0—not knowing others dead due to COVID-19; 1—knowing others dead due to COVID-19); SE: self-experienced COVID-19 illness (0—no; 1—yes); VAC: vaccination (0—no; 1—yes); GHQ-A: GHQ A subscale (anxiety/insomnia); GHQ-B: GHQ B subscale (social dysfunction); GHQ-C: GHQ C subscale (depression); GHQ-D: GHQ D subscale (somatic symptoms). Note: correlation coefficient = Spearman’s ρ. Significance levels: * *p* < 0.05, ** *p* < 0.01, *** *p* < 0.001, **** *p* < 0.0001.

## Data Availability

All research data are available upon request from the corresponding author.

## Abbreviations

The following abbreviations are used in this manuscript:

GHQ-28	General Health Questionnaire
CAS	Coronavirus Anxiety Scale
CiOQ	Changes in Outlook Questionnaire
GAM	Generalized Additive Model
